# Hybrid Feature-Learning-Based PSO-PCA Feature Engineering Approach for Blood Cancer Classification

**DOI:** 10.3390/diagnostics13162672

**Published:** 2023-08-14

**Authors:** Ghada Atteia, Rana Alnashwan, Malak Hassan

**Affiliations:** 1 Department of Information Technology, College of Computer and Information Sciences, Princess Nourah bint Abdulrahman University, P.O. Box 84428, Riyadh 11671, Saudi Arabia; geatteiaallah@pnu.edu.sa; 2 College of Medicine, Alfaisal University, P.O. Box 50927, Riyadh 11533, Saudi Arabia; mhhassan@alfaisal.edu

**Keywords:** leukemia, deep learning, machine learning, feature selection, optimization, classification

## Abstract

Acute lymphoblastic leukemia (ALL) is a lethal blood cancer that is characterized by an abnormal increased number of immature lymphocytes in the blood or bone marrow. For effective treatment of ALL, early assessment of the disease is essential. Manual examination of stained blood smear images is current practice for initially screening ALL. This practice is time-consuming and error-prone. In order to effectively diagnose ALL, numerous deep-learning-based computer vision systems have been developed for detecting ALL in blood peripheral images (BPIs). Such systems extract a huge number of image features and use them to perform the classification task. The extracted features may contain irrelevant or redundant features that could reduce classification accuracy and increase the running time of the classifier. Feature selection is considered an effective tool to mitigate the curse of the dimensionality problem and alleviate its corresponding shortcomings. One of the most effective dimensionality-reduction tools is principal component analysis (PCA), which maps input features into an orthogonal space and extracts the features that convey the highest variability from the data. Other feature selection approaches utilize evolutionary computation (EC) to search the feature space and localize optimal features. To profit from both feature selection approaches in improving the classification performance of ALL, in this study, a new hybrid deep-learning-based feature engineering approach is proposed. The introduced approach integrates the powerful capability of PCA and particle swarm optimization (PSO) approaches in selecting informative features from BPI mages with the power of pre-trained CNNs of feature extraction. Image features are first extracted through the feature-transfer capability of the GoogleNet convolutional neural network (CNN). PCA is utilized to generate a feature set of the principal components that covers 95% of the variability in the data. In parallel, bio-inspired particle swarm optimization is used to search for the optimal image features. The PCA and PSO-derived feature sets are then integrated to develop a hybrid set of features that are then used to train a Bayesian-based optimized support vector machine (SVM) and subspace discriminant ensemble-learning (SDEL) classifiers. The obtained results show improved classification performance for the ML classifiers trained by the proposed hybrid feature set over the original PCA, PSO, and all extracted feature sets for ALL multi-class classification. The Bayesian-optimized SVM trained with the proposed hybrid PCA-PSO feature set achieves the highest classification accuracy of 97.4%. The classification performance of the proposed feature engineering approach competes with the state of the art.

## 1. Introduction

Leukemia is a complex and potentially life-threatening disease affecting individuals of any age group [1]. The disease is characterized by abnormal white blood cells originating in the bone and marrow, spreading throughout the body via the bloodstream. There are two main types of leukemia: acute and chronic. Acute lymphoblastic leukemia (ALL) is particularly aggressive and fast-moving, necessitating early detection for successful treatment [2]. ALL has two main subtypes: B-cell and the T-cell lymphoblastic leukemia. B-cell ALL begins in immature cells that develop into B-cell lymphocytes. On the other hand, T-cell ALL develops in immature cells that would be T-cell lymphocytes. An early leukemia diagnosis is critical for treatment success, and various therapies, such as drug therapy, radiotherapy, and chemotherapy, are available to combat the disease.

However, traditional methods of diagnosing ALL, such as manual blood and bone marrow testing, can be expensive and time-consuming [3]. Computer-aided diagnosis and microscopic image analysis have provided new insights and improved ALL diagnostic accuracy using machine and deep learning [4,5]. By automating the understanding of medical images, researchers can reduce the chances of errors and ensure precise and timely diagnosis. This technology can revolutionize the medical domain by equipping doctors and patients with more efficient and persuasive diagnostic mechanisms and, eventually, more promising results [2,6]. Early detection of leukemia is critical: therefore, an automated diagnosis system can reduce the mortality rate in the medical field at an early stage. Children and adults may die from this disease if not treated promptly, especially with immature bone marrow lymphocytes [7,8]. Various research studies have addressed leukemia detection on blood smear images through diverse techniques for better treatment outcomes. In several studies, machine learning (ML) was employed to classify leukemia in blood images. For instance, Begum et al. [9] developed enhanced hybrid fuzzy C-means with cluster canter estimation for recognizing leukemia WBCs from blood images, separating the nucleus through segmentation. A morphological operations model was used to extract the nucleus, followed by feature extraction to obtain geometric features of the nuclei, then an SVM was trained to classify and predict cancer in blood cell images. The proposed binary SVM classifier achieved sensitivity, accuracy, and specificity values of 81.25%, 89.79%, and 83.27%, respectively. Mahmood et al. [10] utilized four different ML techniques, namely classification and regression trees (CARTs), random forest (RM), gradient boosted machine (GM), and C5.0 decision tree algorithm, to investigate the significance of clinical and phenotypic variables and environmental prerequisites in pinpointing ALL causalities in children. The authors analyzed clinical, phenotypic, and environmental factors to identify causes of child acute lymphoblastic leukemia in 94 pediatric patients, and the best fit was achieved with 99.83% accuracy using classification and regression trees (CARTs). Another study [11] proposed an automated system for detecting acute lymphoblastic leukemia in blood smear images. A pre-processing method and three-phase filtration were introduced to improve segmentation without disrupting leukocyte regions; then, morphological features and statistical features were extracted from those segmented regions. The classification was performed by employing an artificial neural network and the support vector machine. According to the study, both algorithms achieved a specificity of 95.31%, and the sensitivity of the SVM and ANN for classification reached 98.25% and 100%, respectively. 

Deep-learning technology is gaining increasing attention in medical diagnosis for its potential to identify and classify diseases accurately. Many customized deep-learning-based computer-aided diagnosis (CAD) systems have been developed to detect leukemia in images as deep-learning technologies have evolved [12,13]. Rehman et al. [8] proposed a deep-learning-based system to segment cells from stained bone marrow images and classify them as normal or one of the subtypes of ALL. Robust segmentation and convolution, as well as max-pooling layers, were used for training, and a softmax layer was used for image classification. They obtained 97.78% accuracy and outperformed Naïve Bayesian, KNN, and SVM classifiers.

Recently, various pre-trained CNNs, such as ResNet, GoogleNet, DenseNet, Inception, and other CNNs, have been developed and used to automate the medical-image-recognition task. These deep networks were trained using the ImageNet dataset, which is a massive volume of datasets. Pre-trained CNNs can efficiently classify images using transfer learning by fine-tuning the final layers for a related dataset. The research conducted by [14] utilized revolutionary CNN model scaling: multi-attention EfficientNetV2S and EfficientNetB3. Their deep-learning architectures based on pre-trained and a transfer learning-based fine-tuning strategy were used to predict acute lymphoblastic leukemia at an early stage in the medical field. The augmentation techniques were applied on the ISBI-2019 dataset to minimize the error rate during training procedures. The multi-attention EfficientNetV2S and EfficientNetB3 model achieved an accuracy of 99.73% and 99.25%, respectively, for ALL binary classifications. Mondal et al. [15] developed a weighted ensemble deep CNNs model to classify ALL for early diagnosis, including cropping, data augmentation, and rebalancing as pre-processing on the e C-NMC-2019 ALL dataset to increase the network’s generalization. Soft-weighted aggregation combines predictions from different models, i.e., from Xception, VGG-16, DenseNet-121, MobileNet, and InceptionResNet-V2, to perform the final prediction. Model weights were computed based on F1-score, AUC, and Kappa. A weighted ensemble model results with an F1-score of 89.7%, an accuracy of 88.3%, and an AUC of 0.948 and outperforms the best-performing single CNNs model regarding classification performance.

One of the most critical and challenging aspects of applying classification techniques is generating appropriate features. Feature engineering enhances model accuracy by transforming raw data to better represent predictive models. One approach to selecting a subset of functional features from more extensive variables for ALL classifications was through a classification feature selection technique. Almadhor et al. [4] presented a feature extraction technique that used a pre-trained CNN-based deep neutral network to extract the relevant features and analysis of variance (ANOVA), recursive feature elimination (RFE), and random forest (RF) as the feature selection techniques to improve the predictive performance of the model. An ensemble model of KNN, SVM, RF, and NB machine-learning algorithms was proposed and evaluated using the C-NMC leukemia dataset to classify the input image into cancer and healthy cells. The algorithm achieved 87% and 90% accuracy for the ensemble model and SVM, respectively, on ALL binary classifications.

Optimization algorithms are effective in selecting optimal features and hyperparameters for machine-learning models in various applications. Bio-inspired optimization is paramount as an efficient feature reduction tool to produce an accurate medical prediction. However, accurate predictions required suitable hyperparameters settings, and manually tuning these values was still challenging [16,17]. Agustin et al. [18] utilized particle swarm optimization (PSO) to achieve an 87% accuracy rate in classifying ALL patients based on gene expression data. Meanwhile, Sahlol et al. [19] employed the social spider optimization algorithm (SSOA) to select the most relevant attributes using the ALL-IDB acute lymphoblastic leukemia dataset, resulting in a 95% accuracy rate. Bio-inspired optimization algorithms have the potential to improve feature selection in ALL classifications. It has been noticed that metaheuristic optimization algorithms have been utilized mostly for attribute selection from gene expression data. However, only few studies have examined those algorithms for selecting optimal features from image datasets. Further research is necessary to refine these techniques and evaluate their effectiveness on larger datasets [18,19]. In response to this requirement, in this study, we have developed a new hybrid feature engineering approach to select optimal image features for the classification of ALL in BPS images. The proposed approach is based on exploiting pre-trained feature learning with two powerful feature selection approaches, namely principal component analysis (PCA) and particle swarm bio-inspired optimization (PSO). The proposed contributions of this study are listed as follows:

1. Introduction of a new hybrid feature engineering approach based on feature learning extraction and the integration of features generated by PCA and bio-inspired PSO feature selection algorithms for the multi-class classification of ALL in peripheral blood smear images;
2. Incorporation of both feature optimization and Bayesian-based hyperparameter optimization in the introduced approach to boast the classification performance of ALL;
3. Generation of several individually extracted feature sets by pre-trained CNN, PCA, PSO, and integrated PCA-PSO feature set;
4. Comparison of the classification performance of optimized SVM and EL classifiers trained by the individual feature sets and the proposed hybrid feature set.

The rest of the paper is organized as follows: Section 2 describes the image dataset, proposed framework, and methods used in this study; Section 3 depicts the obtained results and the relevant discussion; and Section 4 draws the conclusions.

## 2. Dataset and Methods

This section provides a description of the used leukemia image dataset and the framework proposed in this study. Methods utilized in the introduced framework are also presented in this section.

### 2.1. Dataset

**Figure 1:**
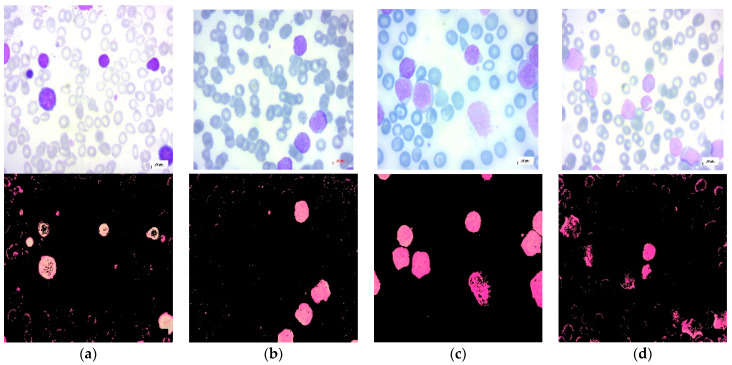
Segmented PBS images from the used dataset [21]: Upper row: original images. Lower row: corresponding segmented images: (**a**) benign; (**b**) early ALL; (**c**) pre-B ALL; (**d**) pro-B ALL.

The dataset used for this study is a public dataset of peripheral blood smear images (PBS) collected from suspected ALL patients [20]. The dataset consists of 3562 images, which are divided into two main classes: benign and malignant. The benign class encompasses hematogones, while the malignant class comprises three malignant lymphoblasts ALL subtypes: early, pre-B, and pro-B acute lymphoblastic leukemia. The dataset images classes are segmented using color-thresholding-based segmentation in the HSV color space [21]. In this study, we considered benign, early, pre-B, and pro-B as four separate classes and built the framework to tackle a multi-class classification problem. The dataset includes 504 benign images, 985 images for the early class, 963 images for the pre-B class, and 804 images for the pro-B class. Figure 1 shows the sample dataset images for each class.

### 2.2. Methods

#### 2.2.1. Framework

**Figure 2:**
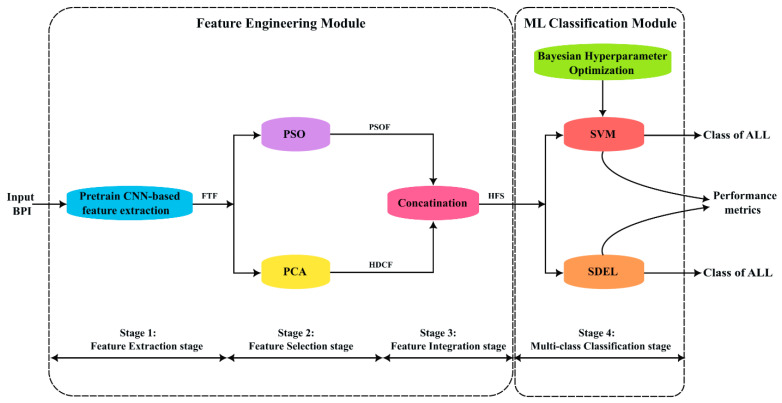
Framework of the proposed feature engineering approach.

The proposed framework of this study comprises two modules and four stages as illustrated in Figure 2. The feature engineering module includes the first, second, and third stages. In the first stage, feature-transfer-based features (FTFs) are extracted from the BPS images by a pre-trained CNN. In the second stage, the PCA and the bio-inspired PSO algorithms are used to select the most effective features from the feature-transfer-based features individually. PCA finds the highest principal components that explain most of the variability in the images; these features are called “HPCF” throughout the paper. On the other hand, PSO searches for the optimal features to be used for classification; the selected features by PSO are called the “PSOF” throughout the paper. A hybrid feature (HFS) set is then constructed by integrating the HPCF and the PSOF in the third stage of the framework. The integrated feature set is then evaluated for the multi-class classification of the ALL by the SVM and ensemble ML classifiers in the fourth stage under the ML classification module. The classification performance of the ML algorithms is evaluated using several performance measures in a 5-fold cross validation scheme.

#### 2.2.2. Feature-Transfer-Based Extraction by GoogleNet

In this work, the GoogleNet pre-trained CNN is used to extract deep image features using feature-transfer learning. GoogleNet is a CNN with a depth of 22 layers arranged into nine inception modules [22]. It is trained on the ImageNet dataset to classify images into 1000 object categories [22]. GoogleNet accepts images of size (224 × 224 × 3). The input images from the dataset are supplied to the CNN after being resized to the designated input size. At the global pooling layer, the pre-trained CNN extracts image features after grouping them over the network layers. The deep features, FTS, of the input images are obtained from the activations of the global pooling layer. The FTS is composed of 1024 features per image. 

#### 2.2.3. Principal Component Analysis

**Figure 3:**
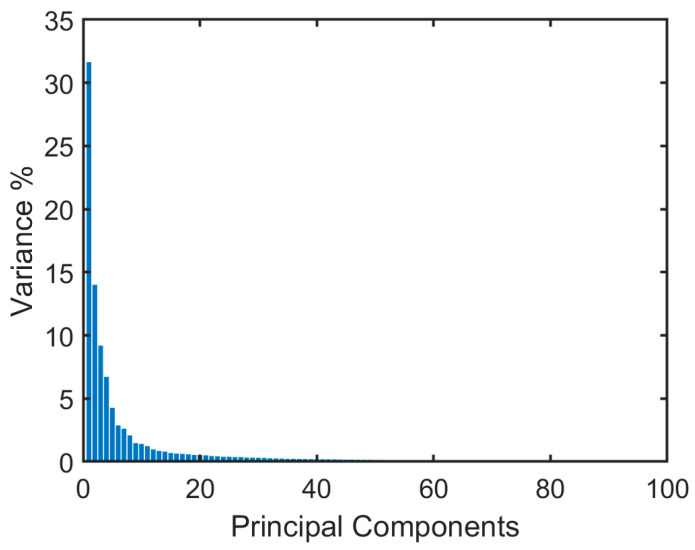
Percent variances per principal component in the HPCF set.

PCA is a classical dimensionality-reduction technique that conserves maximum variation in the data and minimum information loss [23]. It converts the original data features into a new set of linearly uncorrelated features known as principal components (PCs). PCA identifies the directions with the greatest variance within the dataset and then projects the data onto these directions. Principal components are utilized for feature selection, data compression, clustering, and classification and are ranked by the amount of variance they explain. Based on the cumulative percent variance (CPV), the number of derived features, PCs, is determined. CPV is used as a threshold to determine the number of PCs required, covering a specified percentage of the original data. In this study, the PCF set comprises the principal components at CPV of 95%. It was found that 94 PCs explain 95% of the variability in the data. These features form the HPCF set to be used for training the ML classifier. Figure 3 shows the percent variances of the principal components forming the HPCF set. 

#### 2.2.4. Particle Swarm Optimization for Feature Selection

Particle swarm optimization is an evolutionary computation technique that belongs to swarm intelligence methods [24]. PSO is used as an effective tool for optimizing feature selection in machine-learning problems [25]. As with other evolutionary computation (EC) methods, PSO employs a population-based strategy that accommodates the trade-off between feature selection objectives of maximizing classification performance while minimizing the number of features [26]. Each possible solution in PSO is referred to as a bird or particle with no weight or volume that behaves independently in a swarm, while undergoing iterative updates. The ith particle searches in a D-dimensional space for the best solution. The position of this particle is represented by a vector, x_i_ = (x_i1_, x_i2_,..., x_iD_), where x_id_ ∈ [l_d_, u_d_], d ∈ [1,D], l_d_ and u_d_ are the lower and upper boundaries of the d^th^ dimension. The formula for the *i*^th^ particle’s velocity is *v*_i_ = (*v*_i1_, *v*_i2_, …, *v*_iD_). Each particle moves in the direction of the swarm’s global locality (gbest) and its prior best position (pbest) [27]. The ‘pbest’ is the best previous position visited by any particle that is recorded and is called the personal best. However, the best solution visited thus far by the entire swarm is considered the global best, or ‘gbest’. The swarm is initialized with a population of random solutions, and according to the pbest and the gbest, the algorithm seeks the optimal solution by updating the positions and velocities of the particles using Equations (1) and (2), respectively [27]:

(1) $${v_{\mathrm{id}}}^{\left(t+1\right)}=w\times {v_{\mathrm{id}}}^{\left(t\right)}+c_{1}\times r_{1}\times (pbest-{x_{\mathrm{id}}}^{\left(t\right)})+c_{2}\times r_{2}\times (\mathrm{gbest}-{x_{\mathrm{id}}}^{\left(t\right)})$$

(2) $${x_{\mathrm{id}}}^{\left(t+1\right)}={x_{\mathrm{id}}}^{\left(t\right)}+v_{\mathrm{id}}\;$$

where t presents the iteration number of the algorithm, c_1_,c_2_ are acceleration coefficients, and r_1_ and r_2_ are random values uniformly distributed over the interval [0, 1]. The inertia weight, w, balances between the global exploration and local exploitation. The steps followed by the PSO algorithm for selecting the optimum feature set are listed as follows:

Step 1: Initialization of the position and velocity of each particle;

Step 2: Collection of the features selected by a particle;

Step 3: Calculation of the goodness of the particle using the fitness function;

Step 4: Updating the global and local position; i.e., gbest and pbest, respectively;

Step 5: Updating the position and velocity of individual particles; 

Step 6: Terminate if particles pass the minimum fitness threshold; otherwise, go to step 2;

Step 7: Return the best solution with features selected.

**Figure 4:**
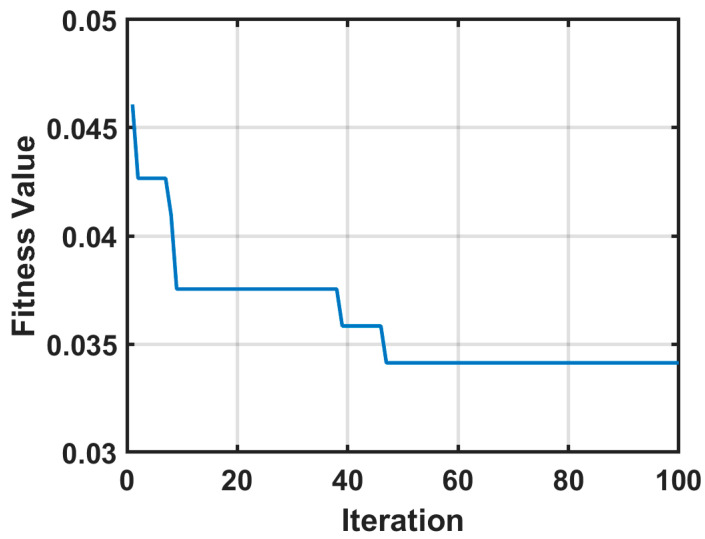
The fitness function performance for the iterations of the PSO run for feature optimization.

In this study, the classification error of the K-nearest neighbor (KNN) classifier with k set to 5 is used to form the fitness function of the PSO algorithm. The PSO algorithm is run over 100 iterations and the fitness value is plotted as in Figure 4. The minimum fitness value was 0.3413, which was recorded from the 47th iteration.

#### 2.2.5. Support Vector Machine (SVM)

A support vector machine or SVM is a nonparametric ML method that is used for nonlinear classification problems. Hyperplanes are defined here for the best class separation with the least amount of error [28]. There are several hyperparameters that should be specified before training the SVM model. Such hyperparameters are the kernel function, kernel scale, and box restrictions. Kernel function determines the kind of nonlinear transformation that would be applied to the input data. The extent to which the kernel significantly fluctuates with the input features is controlled by the kernel scale. The penalty for data samples with significant residuals is determined by the box restriction. In our study, the tuning of the hyperparameters is performed using the Bayesian optimization technique. The optimization process was conducted over 30 iterations with the expected improvement per second plus [29] as the acquisition function of the optimization algorithm.

#### 2.2.6. Subspace Discriminant Ensemble-Learning Classification

Ensemble learning is based on the concept of adopting multiple ML models instead of a single model to resolve nonlinear classification or regression problems. In ensemble learning, the generation and combination of a group of weak learners are adopted to construct a robust prediction model [30]. In our research, the subspace aggregation method is used with discriminant analysis learners to form the EL classifier, or SDEL. This design was selected as it suits the multi-class and multi-predictor problem at hand. The number of learners was set to 30 and the subspace dimension was set to be half of the feature set size, i.e., 47 for HPCF, 262 for PSOF, 512 for the entire feature set, and 309 for the hybrid HFS.

#### 2.2.7. Performance Metrics

The classification performance of the suggested approach is assessed utilizing a 10-fold cross-validation scheme. From the confusion matrix of the classifier, several metrics for performance are computed. The proposed system is evaluated based on its sensitivity (S), specificity (SP), and accuracy (AC). Equations (3)–(5) provide the mathematical expressions for S, SP, and AC, respectively [28,31,32]:

(3) $$S=\frac{TP}{TP+FN}$$

(4) $$SP=\frac{TN}{TN+FP}$$

(5) $$AC=\frac{TP+TN}{TN+TP+FN+FP}$$

where TN represents true negatives, TP represents true positives, FN represents false negatives, and FP represents false positives.

## 3. Results and Discussion

In this investigation, initially, the images of the input dataset were resized and sent to the GoogleNet CNN for the extraction of deep features, the FTF. The FTF set was then sent individually to the PCA and PSO algorithms to generate the HPCF and PSOF sets, respectively. The SVM and ensemble ML algorithms were used for multi-class leukemia classification. Within the proposed framework, four experiments were conducted for the evaluation of the classification performance. In the first experiment, the FTF set was fed merely to the SVM and SDEL classifiers. In the second experiment, the ML classifiers were trained using the PCA features, HPCF. The PSO feature set, PSOF, was utilized for the training of the classifiers in the third experiment and the hybrid feature set, HFS, was used in the fourth experiment. In each experiment, the feature sets were divided into training and testing subsets using a five-fold cross-validation scheme.

**Figure 5:**
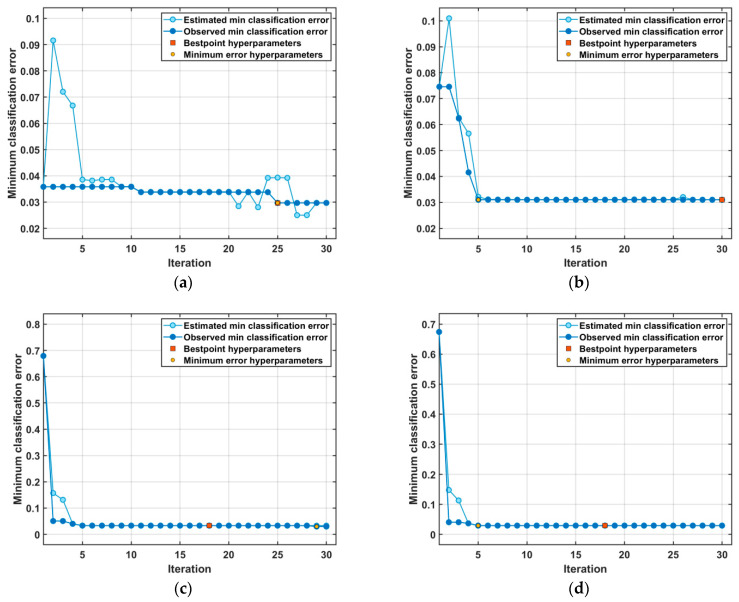
Minimum classification error versus optimization iterations for Bayesian-optimized SVM models trained using: (**a**) FTF set; (**b**) HPCF set; (**c**) PSOF set; (**d**) HFS.

For SVM classification, the Bayesian optimization-based model hyperparameters are depicted in Table 1 for the four experiments. Table 1 also presents the minimum classification error (MCE) for the model with the optimized hyperparameters. Figure 5 illustrates the MCE values recorded by the SVM over the Bayesian optimization run iterations for hyperparameter tuning. Figure 5 shows the MCE plots for the SVM models trained using the FTF, HPCF, PSOF, and HFS sets.

Table 2 depicts the classification performance metrics for the SVM and SDEL classifiers for the four experiments. The weighted accuracy is used to evaluate the classification performance because the classes are imbalanced. Table 2 also presents the feature engineering method, the feature set name, and the number of features included in each set for each experiment. The classification performance of the FTF is considered the baseline performance in this study. The SVM recorded a higher baseline accuracy of 97% than the EL classifier, which recorded 96.5% for the accuracy when trained with the entire FTF set. This performance was maintained for the EL classifier when trained with the 524 features that were selected by the PSO approach. Nonetheless, training the SVM with the PSOF reduces the accuracy to 96.7%. A better accuracy of 96.9% was recorded by the SVM when trained by the 94 PCA-derived features. On the other hand, the SDEL classifier recorded worse performance when trained with the same HPCF set. It is clear from the table that the highest classification accuracy was recorded for both SVM and EN when trained by the PCA-PSO hybrid feature set, which is composed of 816 features. The entries of the feature set that achieved the highest performance are indicated in bold font in Table 2. The SVM achieves the best performance over the SDEL classifier with an accuracy of 97.4%. However, the accuracy of both EN and SVM trained by the proposed hybrid feature set in experiment 4 is still higher than the accuracy achieved by these classifiers when trained by any of the other feature sets in experiments 1, 2, and 3.

In Table 3, we present the classification performance per class for the SVM and SDEL classifiers when trained using the proposed HFS feature set. As leukemia is a life-threatening disease, accurate detection of the disease in the histological blood images is a critical need. Therefore, the sensitivity, SN, and the specificity, SP, are recorded for the benign, early, pre, and pro classes. The sensitivity reveals the capability of the classifier to correctly predict the existence of the disease, while the specificity reveals the classifier’s capability to detect the absence of the disease. The values in Table 3 were calculated by considering the one-versus-all classification approach. It is noticed that the best sensitivity value was recorded for the pro class by both the SVM and SDEL classifiers. The second highest sensitivity was recorded for the early class by the SVM and for the Pre class by the SDEL. The Benign class has recorded the lowest sensitivity by both classifiers. This observation makes sense as the dataset has the lowest number of images for the benign class. The sensitivity of the SVM for the early, pre, and pro classes is higher than that recorded by the ensemble classifier. Nonetheless, both SVM and SDEL recorded the same sensitivity value of 94.1% for the benign class. The highest specificity was recorded for the pro class with the SVM outperforming the EN classifier. The second-best specificity was scored for the benign class equally by the SVM and the EN.

The classification performance of the optimized SVM classifier trained using the proposed feature engineering approach is also assessed against several the state-of-the-art deep-learning-based ALL detectors. In this comparison, we focused on comparing our classifiers performance with that of the systems that use optimization algorithms for feature selection developed for the classification of leukemia although the datasets used in these studies are different. Table 4 compares our system with recent leukemia diagnosis CAD systems in terms of the used feature engineering approach, the type of classifier, and the classification accuracy. The proposed approach yields higher classification accuracy with respect to all other classifiers that employs other feature-optimization techniques. In general, the comparison reveals that the introduced Bayesian-based optimized SVM trained by the PSO-PCA feature engineering-based approach achieves superior classification performance over existing feature-optimization-based deep-learning systems developed for leukemia diagnosis. 

## 4. Conclusions

In this study, a hybrid approach to feature engineering based on deep learning is proposed. First, the feature-transfer capability of the GoogleNet convolutional neural network (CNN) is used to extract image features. PCA is used to generate a feature set of the principal components that accounts for 95% of the data’s variability. Optimal image features are also collected by the PSO algorithm. The PCA and PSO-derived feature sets are then combined to produce a composite set of features, which is used to train a Bayesian-based optimized support vector machine (SVM) and a subspace discriminant ensemble-learning (SDEL) classifier. The classification performance of optimized SVM and EL classifiers are examined when trained by all extracted features, PCA-derived, PSO-derived, and the hybrid PCA-PSO feature set. The SVM classifier shows better classification performance than the EL for all types of feature sets. Both the SVM and EL show better prediction performance when trained by the proposed hybrid feature set than when trained with the other feature sets. The obtained results depict that ML classifiers present the highest sensitivity and specificity for the pro class and the least values for the benign class. Moreover, the classification performance of the introduced Bayesian-optimized SVM classifier trained by the proposed PSO-PCA hybrid feature engineering approach outperforms the state of the art. The proposed approach takes advantage of three up-to-date technologies: feature-learning of pre-trained CNNs, PSO, and PCA approaches of feature selection. The proposed hybrid approach provides a feature set that boasts the classification performance of ML classifiers over that trained by the original sets generated individually by the GoogleNet, PCA, and PSO algorithms for ALL diagnosis in BPI. Although the proposed approach provides an improved performance for ALL detection, the classification performance in other diseases should be checked. This work will be extended in the future by employing other metaheuristic feature-optimization algorithms and by investigating the classification performance of ML classifiers for leukemia and other disease diagnosis.

## Figures and Tables

**Table 1:** SVM models hyperparameters selected by the Bayesian optimization approach.

Experiment	Feature Engineering Approach	Kernel Scale	Kernel Function	Box Constrain	Classification Error
Experiment 1	Feature Transfer (GoogleNet)	1	Quadratic	0.2856	0.0296
Experiment 2	PCA	1	Quadratic	977.59	0.031
Experiment 3	PSO	1	Quadratic	0.001017	0.033
Experiment 4	Integration of PCA and PSO Features	1	Quadratic	981.0134	0.0289

**Table 2:** Classification performance of the SVM and SDEL classifiers in the four experiments. Entries of the highest classification performance are indicated in bold font.

Experiment	Feature Engineering Approach	Feature Set Name	# Features	SVM AC%	SDEL AC%
Experiment 1	Feature Transfer (GoogleNet)	FTF	1024	97	96.5
Experiment 2	PCA	HPCF	94	96.9	95.4
Experiment 3	PSO	PSOF	524	96.7	96.5
Experiment 4	Integration of PCA and PSO Features	HFS	618	97.4	96.8

**Table 3:** Sensitivity (S) and specificity (SP) per class of the ML classifiers trained by the hybrid PSO-PCA feature set.

Class	SVM		SDEL	
	S%	SP%	S%	SP%
Benign	94.1	98.9	94.1	98.9
Early	97.4	98.8	96.7	98.6
Pre-B	97.7	98.6	96.8	98.6
Pro-B	99.0	99.8	98.8	99.6

**Table 4:** Comparison results of the proposed Bayesian-optimized SVM trained using the PSO-PCA feature-engineering-based approach with the state-of the-art leukemia-detection systems.

Feature Engineering Approach	Classifier	Optimization	AC%	Publication
Feature extraction: VGGNet. Features selection: salp swarm optimization.	KNN, SVM, decision tree, naive Bayes	Salp Swarm Optimization	96.1	[33]
Feature extraction: Hand-crafted features from input images. Features selection: social spider optimization.	Ensemble of classical ML classifiers	Social Spider Optimization	95.2	[19]
Feature extraction: customized CNN. Features selection: grey-wolf-based Jaya optimization.	Customized CNN	Grey-wolf-based Jaya Optimization	93.5	[34]
Feature extraction: pre-trained CNNs. Features selection: competitive swarm optimization.	ABiLSTM	Competitive Swarm Optimization	96	[35]
Feature extraction: GoogleNet. Features selection: hybrid PSO-PCA approach.	Bayesian-optimized SVM	Hybrid PSO-PCA approach	97.4	Proposed study

## Data Availability

Data source and details are provided in the Dataset and Methods section.
